# Investigating the growing trend of non-drinking among young people; analysis of repeated cross-sectional surveys in England 2005–2015

**DOI:** 10.1186/s12889-018-5995-3

**Published:** 2018-10-10

**Authors:** Linda Ng Fat, Nicola Shelton, Noriko Cable

**Affiliations:** Department of Epidemiology and Public Health, 1-19 Torrington Place, London, WC1E 7HB UK

**Keywords:** Non-drinking, Alcohol consumption, Young people, Adolescents, Trends

## Abstract

**Background:**

Non-drinking among young people has increased over the past decade in England, yet the underlying factor driving this change is unknown. Traditionally non-drinking has been found to be associated with lower socio-economic status and poorer health. This study explores among which sub-groups non-drinking has increased, and how this correlates with changes in drinking patterns, to identify whether behaviours are becoming more polarised, or reduction is widespread among young people.

**Methods:**

Among participants aged 16 to 24 years (*N* = 9699), within the annual cross-sectional nationally-representative Health Survey for England 2005–2015 datasets, the following analyses were conducted: 1) The proportion of non-drinkers among social-demographic and health sub-groups by year, and tests for linear trends among sub-groups, adjusting for age were calculated. In pooled analyses, an interaction between year and each variable was modelled in sex- and age-adjusted logistic regression models on the odds of being a non-drinker versus drinker 2) At the population level, spearman correlation co-efficients were calculated between the proportion non-drinking and the mean alcohol units consumed and binge drinking on the heaviest drinking day, by year. Ordinary least squares regression analyses were used, modelling the proportion non-drinking as the independent variable, and the mean units/binge drinking as the dependent variable.

**Results:**

Rates of non-drinking increased from 18% (95%CI 16–22%) in 2005 to 29% (25–33%) in 2015 (test for trend; *p* < 0.001), largely attributable to increases in lifetime abstention. Not drinking in the past week increased from 35% (32–39%) to 50% (45–55%) (*p* < 0.001). Significant linear increases in non-drinking were found among most sub-groups including healthier sub-groups (non-smokers, those with high physical activity and good mental health), white ethnicity, north and south regions, in full-time education, and employed. No significant increases in non-drinking were found among smokers, ethnic minorities and those with poor mental health. At the population-level, significant negative correlations were found between increases in non-drinking and declines in the mean units consumed (ρ = − 0.85, *p* < 0.001), and binge drinking (ρ = − 0.87, *p* < 0.001).

**Conclusion:**

Increases in non-drinking among young people has coincided with a delayed initiation into alcohol consumption, and are to be welcomed. Future research should explore attitudes towards drinking among young people.

**Electronic supplementary material:**

The online version of this article (10.1186/s12889-018-5995-3) contains supplementary material, which is available to authorized users.

## Background

Abstention from alcohol or ‘non-drinking’ has risen in Great Britain. Around 10% classified themselves as non-drinkers in 1998, increasing to 15% in 2009 [1], with a further increase to 21% in 2013 [2]. The increase has been the greatest among young adults. The proportion of non-drinkers (including those who had not had a drink in the past year) among those aged 16–24 doubled from 12% in 2011 to 24% in 2014 in England [3]. This phenomenon among young people has received much media attention, with some referring to it as *“The Rise of the Teetotal generation”* [4]. Whilst the media has speculated on the causes, such as greater health concerns, to our knowledge this has not been investigated formally.

Research has established a social gradient in non-drinking. Non-drinkers are more likely to have lower education, lower income, live in the most deprived areas, and be unemployed compared with those who drink alcohol in moderate amounts [5–9]. Furthermore, poor health being a reason for abstaining are well established [9–13]. However, it is possible that increases in abstention may be due to health promoting reasons, encouraged by health promoting campaigns such as ‘Dry January’ [14], and the emerging evidence of the link between any alcohol consumption and the risk of cancer [15], and obesity [16]. A recent study on mental wellbeing found that being a non-drinker was associated with increased chances of both high and low mental wellbeing (versus medium wellbeing), compared with being a moderate drinker [17], suggesting that non-drinkers may have polarised characteristics in terms of health.

The aim of this research is to identify whether increases in non-drinking have occurred among factors commonly associated with non-drinking such as low socio-economic position and poorer health, versus non-traditional factors such as among healthier groups or higher social status. Drinking habits are defined from an early age which can impact health later on in life [18], so it is important to understand how drinking behaviours are changing among this age-group. Another aim of this work is to identify how changes in non-drinking among young people has accompanied changing drinking patterns overall, for example whether behaviour is becoming more polarised between none and heavy drinking, which could be problematic.

### Research questions

In our study, we address the following research questions, among 16–24 year olds in England, from 2005 to 2015:

1. How has non-drinking increased? Is this pattern observed for the different types of non-drinkers; lifetime abstainers, ex-drinkers, occasional drinkers? In addition, for periodic abstinence (those who abstain in the previous week)?
2. Among which social/demographic and health subgroups has non-drinking increased?
3. In multivariable analyses, have the chances of being a non-drinker increased more strongly for any particular sub-group over-time?
4. How do changes in the proportion of non-drinking over time, correlate with changes in mean consumption, and heavy episodic drinking over time?

## Methods

### Study design and participants

This study uses the Health Survey for England (HSE) 2005 to 2015, accessed via he UK Data Service, subject to their end user license [19]. The Health Survey for England is a nationally-representative annual cross-sectional survey of the population living in private households in England [19]. Participants were selected using multi-stage stratified-sampling; selecting participants within primary sampling unit (PSUs). Household response rates declined somewhat over the period, ranging from 74% in 2005 to 60% in 2015 [20]. Non-response weights have been calculated and were included in the datasets. Ethical approval for the HSE was obtained ahead of the data collection from the relevant ethics committee [21], data was anonymised and further ethical approval was not needed. In our study, the sample was limited to the participants aged 16 to 24 years, who answered questions about drinking status (*N* = 9699) in surveys between 2005 and 2015. Non-response to the drinking status question accounted for 1.5% of all 16 to 24-year olds. Information was collected via trained interviewers who administered the interview face-to-face in participants’ households using CAPI and a self-completion booklet.

### Variables

#### Non-drinking

Non-drinkers were defined as participants who reported ‘no’ to the question on drinking status: *“Do you ever drink alcohol nowadays, including drinks you brew or make at home?”* Lifetime abstainers; non-drinkers who reported they had always been a non-drinker, and former drinkers; non-drinkers who reported they had not always been a non-drinker, were derived from a follow-up question specific to non-drinkers.. Non-drinkers were also asked if they drank occasionally, who we refer to as ‘occasional drinkers’. Non-drinkers have been found to be a heterogeneous group, consisting of lifetime abstainers, former drinkers and occasional drinkers [22]. In addition, to explore periodic abstinences, we also examined changes in the prevalence of not having an alcoholic drink in the past week.

#### Drinking patterns

Drinking patterns were identified based on alcohol units drank on the heaviest drinking day in the past week. These questions were asked consistently across the survey years between 2005 and 2015. A category for drinkers, drinking alcohol within limits at the time of the survey (not exceeding 4 units for men, and 3 units for women on any day [23]) were created. Binge drinking was defined as drinking twice the recommended daily limits on the heaviest drinking day.

#### Social and demographic variables

The following variables were considered as sub-groups; sex, broad ethnicity (white/non-white), full-time education versus not in full-time education, north and south regions of England, area-deprivation, measured by the Index of Multiple Deprivation (IMD) in quintiles dichotomised (three least deprived versus two most deprived area), urban location (urban/town/village), household level national-statistics socio-economic classification (NS-SEC) (managerial professional/intermediate/routine manual) and individual employment status (employed/non-employed).

#### Health and health behaviours

Positive health behaviours and health statuses were considered including non-smokers (versus smokers), eating five or more portions of fruit and vegetable a day (versus 3–4 or 0–2 portions), high physical activity (versus medium or low level), and up to normal Body Mass Index (BMI) category (underweight/normal (up to 24.9 kg/m^2^), overweight or over (25 kg/m^2^ or over). Apart from objectively collected data on BMI, all information was self-reported. Physical activity was measured using the short-form International Physical Activity Questionnaire (IPAQ) [24], which has been asked annually since 2013. Questions on fruit and vegetable consumption were not asked in 2012 and 2014; all other years were presented. The proportion of non-drinkers among those with no longstanding illness (versus those with a longstanding illness or limiting longstanding illness) were also explored. Mental health and wellbeing was measured through the 12 item General Health Questionnaire (GHQ-12), and the Warwick-Edinburgh Mental Wellbeing scale (WEMWBS), respectively. Total GHQ-12 scores were calculated by assigning values of 0 if symptoms were not present, or 1 if symptoms were present on each of the 12 items, and summing scores on the items together (maximum score 12). We dichotomised total GHQ-12 scores into zero (no evidence of mental illness), or 1 or more (less than optimal mental health including probable mental ill health) [25]. GHQ-12 scores were not collected in 2007, 2011, 2013 and 2015. Participants with total scores on the 14-item WEMWBS with five response categories (scored zero to five), ranging from 14 to 70 were dichotomised. Participants with scores one standard deviation below the mean were categorised as having low mental wellbeing (14–42), versus above one standard deviation from the mean (mid to high wellbeing; 43 or higher) [17]. Questions from the WEMWBS scale have been asked annually since 2010. The GHQ-12 and WEMWBS were administered via a self-completion booklet, which has a higher non-response rate.

#### Statistical analyses

All analyses applied complex survey design and non-response weighting. The proportion of non-drinkers among the population and corresponding confidence intervals were calculated for each year from 2005 to 2015. Significant differences were highlighted when proportions differed from the 2005 start year. Tests for linear trends in the level of non-drinking over time, were examined for each sub-group using regression analyses, modelling year as an independent variable and non-drinking as the dependent variable and adjusting for age. Trends were illustrated in charts using three-year moving averages. The same analyses were repeated among different social-demographic and health sub-groups. Information for variables with missing year’s data, were modelled as consecutive years, observing whether a significant linear increase was found among the years that data was collected.

In pooled analyses of all datasets, we examined whether the chances of being a non-drinker increased greater by year for certain sub-groups, by conducting logistic regression on the odds of being a non-drinker versus drinker, modelling an interaction effect between each sub-group and year, adjusting for age and sex. These analyses were limited to variables that had information on all years; urban area, IMD, educational, employment, household social class, smoking status, limiting longstanding illness statuses which was dichotomised (BMI was not included due to a relatively high proportion of missing BMI measurements (14%)). In preliminary analyses, the interaction effect between broad ethnic groups (white vs. non-white) and year was significant (OR = 1.06 (95%CI 1.01–1.11) *p* = 0.03), suggesting that the odds of being a non-drinker have increased faster for the white than non-white population. However, in models there were large effect sizes, due to sparse data problems [26]. Therefore we limited these logistic regression models to white-participants only (*N* = 7934).

We examined whether increases in non-drinking were related to changes in drinking patterns among young people by undertaking ecological analyses. Spearman correlation co-efficient were calculated between the proportion of non-drinkers by year and the proportion binge drinking, and mean units consumed on the heaviest drinking day. Ordinary least squares regression analyses were used to test the strength and direction of the relationship between the proportion non-drinking (independent variable) and the proportion binge/mean units (dependent variable), over time. The relationship is illustrated using scatter diagrams. As a sensitivity analyses we also examined the relationship between the proportion of non-drinkers and the proportion binge drinking and mean units consumed on the heaviest drinking among drinkers only, which does not include the numbers of non-drinkers in its calculation.

## Results

### Descriptive trend analyses

**Fig. 1:**
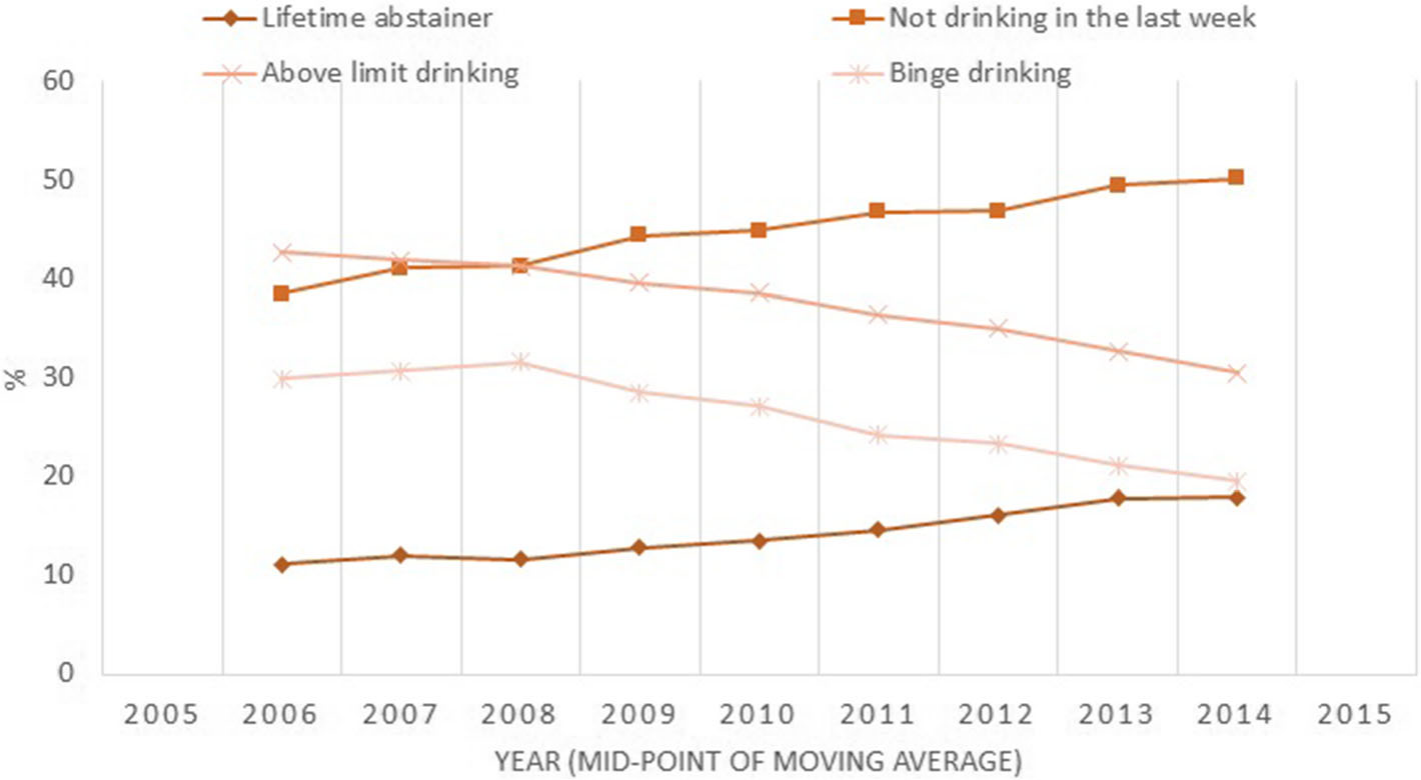
Three-year moving average in drinking pattern among 16 to 24 year olds, HSE 2005-2015

Among those aged 16 to 24 years, the proportion of non-drinkers increased from 18% (95% CI 16–22%) in 2005 to 29% in 2015 (CI 25–33%) (test for linear trend *p* < 0.001, Table 1, see Additional file 1: Table S1 for confidence intervals)). The increase was largely attributable to an increase in the proportion of lifetime abstainers (9% (CI 7–11% to 17% (CI 13–21%, *p* < 0.001), rather than ex-drinkers (2% (CI 1–3%) to 2% (CI 1–4%), *p* = 0.371). There were also increases in the proportion whom had not drunk any alcohol in the last week, from 35% (CI 32–40%) in 2005 to 50% (CI 45–55%) in 2015 (*p* < 0.001), and from 22% (CI 19–26%) to 33% (CI 28–37%) among drinkers only (*p* < 0.001). There were significant decreases in the proportion who drank above limits (43% (CI 38–47% to 28% (CI 24–32%), *p* < 0.01), or binge drank (27% (CI 23–31%) to 18% (15–22%), *p* < 0.001) but no differences in the proportion drinking within limits (22–22%. *p* = 0.258). These trends have been depicted as three-year moving averages in Fig. 1.

**Table 1:** Trends in the proportion (%) non-drinking and drinking pattern among 16–24-year olds, HSE 2005–2015^a^

	Year											N	*p*-value trend	% Change 2005–2015
	2005	2006	2007	2008	2009	2010	2011	2012	2013	2014	2015			
Types of non-drinkers														
All	18	23	18	23	24	26	23	**27**	**28**	**31**	**29**	9699	*p* < 0.001	61
Lifetime abstainer	9	14	10	12	13	14	14	**16**	**18**	**19**	**17**	9699	*p* < 0.001	89
Ex-drinker	2	2	1	2	2	2	2	2	2	2	2	9699	0.371	0
Occasional drinker	7	7	7	8	9	10	8	9	8	9	10	9699	0.028	43
Not drinking in the last week														
Not drinking in the last week	35	42	39	43	**42**	**48**	**45**	**48**	**48**	**52**	**50**	9489	*p* < 0.001	43
Not drinking in last week (drinkers only)	22	25	27	28	26	31	**29**	**30**	**30**	**34**	**33**	7152	*p* < 0.001	50
Drinking on heaviest drinking day														
Within limits	22	16	19	17	17	14	18	18	19	17	22	9489	0.258	0
Above limit	43	43	**43**	**40**	**41**	**38**	**37**	**34**	**33**	**31**	**28**	9489	*p* < 0.001	−35
Binge	27	29	33	29	32	24	25	**23**	**22**	**19**	**18**	9489	*p* < 0.001	−35

^a^Figures in bold indicate statistical significantly different to 2005, for 95% confidence intervals see Additional file 1: Table S1

### Sub-groups analyses

Examining the level of non-drinking by social and demographic sub-groups (Table 2, see Additional file 1: Table S2 for confidence intervals), linear increases in the prevalence of non-drinking by year from 2005 to 2015 were found among males, females, those aged 16–17, 18–24 years, white ethnicity, in full-time education and those living in urban, town/villages, north and southern regions of England, areas along the five deprivation quintiles, among those employed and non-employed, and all household level occupational groups (Table 2) (*p* < 0.05). In 2005, just over a quarter, 28% (CI 22–35%) of 16 to 17 year olds were a non-drinker, by 2015 this had increased to just under a half (48%, CI 39–56%). Likewise the proportion of non-drinkers increased among 18 to 24 years olds from 15% in 2005 (CI 12–18%) to 24% (CI 20–29%) in 2015. Among the white population, non-drinking increased from 14% (12–17%) in 2005 to 20% (17–24%) in 2015. The proportion of non-drinkers among the employed doubled from 2005 (9%, CI 7–13%) to 2015 (18%, CI32–45%). No significant increases were found among non-white minorities (*p* = 0.421).

**Table 2:** Trends in the proportion (%) of non-drinkers among social and demographic sub-groups, HSE2005–2015^a^

	Year											N	*p*-value trend	% Change 2005–2015
	2005	2006	2007	2008	2009	2010	2011	2012	2013	2014	2015			
All	18	23	18	23	24	**26**	23	**27**	**28**	**31**	**29**	9699	*p* < 0.001	61
Age group														
16–17	28	31	26	38	42	33	38	**44**	**44**	**50**	**48**	2294	*p* < 0.001	71
18–24	15	21	16	17	18	24	20	**23**	**23**	**25**	**24**	7405	*p* < 0.001	60
Sex														
Males	16	24	14	21	20	25	20	**26**	**27**	**29**	25	4368	*p* < 0.001	56
Females	20	23	23	24	28	27	27	28	29	**32**	**33**	5331	*p* < 0.001	65
Ethnicity														
White	14	13	11	15	15	18	17	19	17	**23**	**20**	8168	*p* < 0.001	43
Non-white	54	72	49	59	69	61	57	63	67	66	68	1531	0.421	26
Region														
North	17	21	19	18	28	23	23	21	29	**31**	**30**	3258	*p* < 0.001	76
South	19	24	18	25	22	27	24	29	**28**	31	28	6441	*p* < 0.001	47
Urban/Rural locality														
Urban	19	25	20	24	26	27	24	**29**	**29**	**31**	**32**	7871	*p* < 0.001	68
Town/Village	16	14	9^a^	13	12	21	23	16	19	**28**	**26**	1828	*p* < 0.001	63
Area deprivation														
Least deprived	13	17	15	16	19	19	18	**21**	20	**24**	**23**	5205	*p* < 0.001	81
Most deprived	25	31	22	30	30	34	32	35	37	**38**	36	4494	*p* < 0.001	42
Household social class														
Managerial and Professional	16	18	12^b^	16	18	20	20	20	**23**	**25**	**25**	3168	*p* < 0.001	54
Intermediate	17	17	18	25	35	30	28	22	24	**33**	**33**	1805	*p* < 0.001	94
Routine manual	19	27	21	25	23	28	24	**32**	**33**	**34**	25	3938	0.001	28
Educational status														
Full-time education	23	29	25	28	26	30	27	33	33	**38**	36	4360	*p* < 0.001	57
Not in FTE	14	18	12	17	22	21	20	22	**23**	**23**	**23**	5326	*p* < 0.001	64
Employment status														
Employed	9	13	9	14	17	17	13	15	**19**	**19**	**18**	4103	*p* < 0.001	100
Unemployed	26	32	26	29	28	31	30	34	34	**39**	**38**	5552	*p* < 0.001	46

^a^Figures in bold indicate statistical significantly different to 2005, for 95% confidence intervals see Additional file 1: Table S2

^b^Alternative Baseline year, where no difference found with 2005

Examining the level of non-drinking by health behaviours (Tables 3, 95% CI (Additional file 1: Table S3); linear increases in non-drinking were found among non-smokers, those with normal weight, and those eating three to four portions of fruit and vegetables per day (*p* < 0.001). In 2005, 23% (CI 19–28%) of non-smokers were non-drinkers by 2015, this had risen to 34% (CI 29–39%). Among those eating three to four portions of fruit and vegetables, the proportion of non-drinkers increased by 91%, from 2005 (17% (CI 13–22%) to 2015 (32% (CI 25–40%). Linear increases in the proportion of non-drinkers were also found among those classified as overweight or above (BMI ≥ 25), and those eating none to two proportions of fruit and vegetables a day (*p* < 0.001). There were no statistical significant increases in the proportion of non-drinkers among smokers (*p* = 0.083), and those consuming more than five fruit or vegetables per day (*p* = 0.084). From 2013 to 2015, there was a linear increase in the proportion of non-drinkers among those with high physical activity levels (*p* = 0.039), but no significant increase was found for those with low to medium physical activity.

**Table 3:** Trends in the proportion (%) of non-drinkers among health and health behaviour sub-groups, HSE2005–2015^a^

	Year											*N*	*p*-value trend	% Change 2005–2015
	2005	2006	2007	2008	2009	2010	2011	2012	2013	2014	2015			
Health Behaviours														
Smoking status														
Non-smokers	23	28	22	27	28	31	27	**34**	**33**	**36**	**34**	6393	*p* < 0.001	50
Smokers	12	14	10	13	13	15	15	11	15	17	16	3257	0.083	36
BMI category														
Normal/underweight	17	23	19	22	26	25	21	**28**	**27**	**30**	26	5523	*p* < 0.001	55
Overweight/obese	17	22	15	21	17	22	25	21	24	**29**	**29**	2976	*p* < 0.001	74
Fruit and vegetable consumption categories														
Five or more portions of fruit and veg	20	24	18	24	29	27	23	–	25	–	26	2494	0.084	30
Three to four	17	23	20	22	21	25	25	–	**33**	**–**	**32**	2843	*p* < 0.001	91
0–2	18	23	16	22	21	26	22	–	26	–	28	2863	0.002	55
Physical Activity														
Low	–	–	–	–	–	–	–	–	43	39	35	1496	0.149	−19
Medium	–	–	–	–	–	–	–	–	25	30	30	1628	0.142	19
High	–	–	–	–	–	–	–	–	15	23	22	1706	0.039	44
Health														
GHQ score														
Zero	20	21	–	24	20	27	–	**28**	**–**	**37**	–	3619	*p* < 0.001	79
One or more (less than optimal)	14	23	–	20	27	22	–	23	–	22	–	2638	0.174	54
WEMWBS score														
Mid to high wellbeing	–	–	–	–	–	24	22	24	26	30	29	3482	0.006	18
Low wellbeing	**–**	**–**	**–**	**–**	**–**	23	23	24	30	29	27	2477	0.237	19
Longstanding illness (LSI)														
No longstanding illness	18	23	19	23	22	24	23	**27**	**28**	**31**	**30**	7676	*p* < 0.001	67
Longstanding illness	20	25	13^b^	23	29	33	24	27	26	**30**	25	2019	0.02	25

^a^Figures in bold indicate statistical significantly different to 2005, for 95% confidence intervals see Additional file 1: Table S3

^b^Alternative Baseline year, where no difference found with 2005

Examining the level of non-drinking by health; a linear increase in non-drinking was found among those with a longstanding illness (18% (CI 14–21%) in 2005 to 30% (CI 25–35%) in 2015) as well as without a longstanding illness (13% (CI 8–20%) in 2007 to 30% (CI 22–38%) in 2014) (*p* < 0.05). A linear increase in non-drinking was also found among those with normal GHQ score of zero (no evidence of mental ill health), and mid-to-high mental wellbeing (*p* < 0.001), but not for those with low mental wellbeing (*p* = 0.237) or less than optimal mental health (GHQ score ≥ 1) *p* = 0.258.Among those with normal GHQ scores, the proportion of non-drinkers increased from 20% (CI 17–25%) in 2005 to 37% (31–43%) in 2014.

### Interaction between year and sub-groups in pooled regression analyses

Limiting to white participants only, for every year increase, the odds of being a non-drinker versus drinker increased by 7% (OR = 1.07 95% Confidence interval 1.04–1.09), after adjusting for all variables. There were no significant interactions between year and any of the variables, after adjusting for age and sex (Additional file 1: Table S4).

### Ecological analyses among the population; correlations between non-drinking and heavy episodic drinking/mean units

**Fig. 2:**
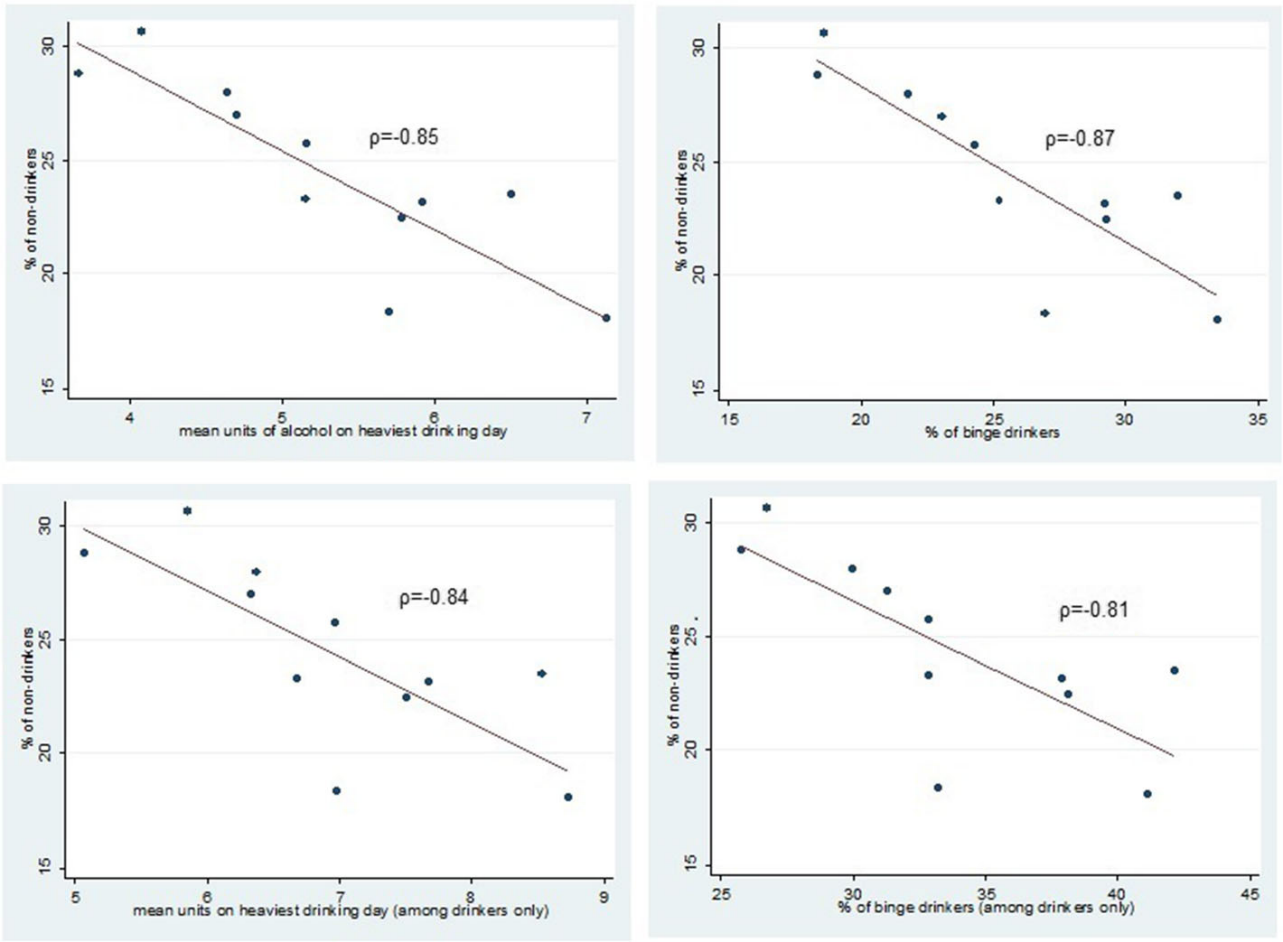
Scatter diagram showing the relationship between the proportion non-drinking and proportion binge drinking/mean units consumed, HSE 2005-2015

Correlations between the proportion of non-drinkers by year, and the mean units of alcohol consumed on the heaviest drinking day, and proportion binge drinking was negative (mean units ρ = − 0.85, binge ρ = − 0.87, *p* < 0.001, Table 4). Interpreting the regression co-efficient; a one percentage point increase in non-drinking among 16 to 24 year olds, predicted a 0.22 reduction in mean units consumed on the heaviest drinking day (95% CI -0.32—0.12), and a 1.06 percentage point decrease in proportion binge drinking (95%CI 1.56—0.54) in the total population. The co-efficient did not change dramatically when using mean units and proportions binge drinking limited to drinkers only (− 0.20 unit reduction (− 0.34—0.07), − 1.00 percentage point reduction (95%CI -1.68- -0.31). The direction of the association is illustrated Fig. 2.

**Table 4:** Correlation and regression co-efficient between the proportion non-drinking over time, and proportion binge drinking / mean unit of alcohol consumed on the heaviest drinking day among 16–24 year olds, HSE2005–2015

	Spearman correlation coefficient	*p*-value	Regression coefficient	(95% CI)
Outcome variable				
Proportion Binge	−0.87	*p* < 0.001	−1.06	(−1.56--0.54)
Mean units	−0.85	*p* < 0.001	−0.22	(−0.32--0.12)
Outcome variable (calculated among drinkers only)				
Proportion Binge	−0.81	0.03	−1.00	(−1.68- -0.31)
Mean units	−0.84	*p* < 0.001	− 0.20	(− 0.34--0.07)

## Discussion

Identifying as a non-drinker has increased among young people, mainly attributable to fewer younger people taking up drinking, shown by the rate of lifetime abstainers almost doubling from 9% in 2005 to 17% in 2015. In addition more drinkers were engaging in weekly abstinence. In 2005, just over a third of those aged 16 to 24 did not have a drink in the past week, compared with a half in 2015. Previous research has identified stigma associated with non-drinking [27]. However, non-drinking appears to have increased across almost all sub-groups, including healthy groups (non-smokers, high physical activity and good mental health), the white population, those in employment or full-time education, across household NS-SEC group and among all levels of area-deprivation, and both northern and southern regions. Whilst traditionally poorer health and lower socio-economic status has been associated with non-drinking [9, 28], this might suggest that the norms around non-drinking are changing, and this behaviour is becoming more mainstream among young people. Furthermore, no increasing trends in non-drinking were found among variables commonly associated with non-drinking, such as among ethnic minorities, and those with poorer mental health [9, 28]. We cannot know the reasons for why non-drinking has remained stable among ethnic minorities. Non-drinking remains higher among ethnic minorities than the white population.

Increasing rates of non-drinking among young people are to be welcomed. Instead of behaviours becoming polarised between extremes such as binge drinking and abstinence, increases in non-drinking were correlated with a decline in mean units of alcohol consumed, and the proportion binge drinking. This is consistent with population theories, including Skog’s the collectivity of drinking cultures [29, 30], which suggests that it is the average drinker which influences heavy and problematic drinking [29, 31–34]. More young people not drinking may influence lower average consumption overall, which tends to reduce problematic drinking.

Declines in alcohol consumption among young people have been found across other high income countries, including within North America and Europe [34, 35], although in Canada rates of binge drinking increased from 1996 to 2013 [36]. It is difficult to pinpoint a single factor that has caused the decline in alcohol consumption. Policies in England coinciding with declines including tougher and stricter licensing laws on the sales of alcohol to those aged under 18 years, which is illegal [37]. In 2005 three in ten 16 to 17 year olds reported being a non-drinker increasing to nearly one in two in 2015. Much of the increase has come from young people not taking up alcohol at all, indicating that initiation into alcohol has been delayed. Around 39% of those aged 11–13 never had an alcohol drink in 2003, compared with 48% in 2010 [38]. Among a cohort of 10 to 15 year olds, happiness and awareness of alcohol-harm was associated with not being initiated into alcohol use [39]. Increasing awareness of the harms of alcohol may have played an important role in decreasing alcohol consumption among young people and the general population [3]. Indeed this would correlate well with the increase in non-drinking among healthier sub-groups, although we also found trends in less healthier sub-groups (e.g. overweight/obese, eating zero to two proportions of fruit and vegetables a day), suggesting that there may be other factors underpinning the increase in non-drinking.

The non-significant associations between year and social and health variables, suggests that the increase is not attributable to any one factor, and the causes are likely to be multi-factorial or cultural. There were factors not explored in this study such as media use, which might be changing the way young people spend their leisure time. Further qualitative research is required to analyse attitudes towards drinking among young people and how they may have changed, including changes in life priorities and parental supervision in relation to alcohol use. The relationship between increases in non-drinking among students and the employed may be due to increased work stress or pressure, however we cannot know this from this data, further we did not find increases in non-drinking among those with poor mental health or wellbeing. This needs to be investigated further. Factors influencing the shift away from drinking (and subsequently problematic drinking), could be capitalised on to ensure that sensible drinking continues to be encouraged. Whilst rates are falling, young people remain the most likely group to be binge drinking [3]. Heavy episodic drinking increases the risk of alcohol-related harm, such as crime and accident and emergency attendance, which places considerable burden on the National Health Service [40, 41]. Rates have been falling from a very high level, where a third of young people were found to be binge drinking in 2002 and 2007 [3]. Efforts to reduce problematic drinking should not be ignored. Furthermore, rates of non-drinking have not increased among smokers, suggesting that these risky behaviours continue to cluster [42, 43], and there may be subsets of young people with very unhealthy behaviour. Alcohol or cigarette consumption are likely to be gateways into each other [44]. Targeting these behaviours in tandem, could have positive implications for public health overall.

### Strengths and limitations

Strengths of this study include the use of a large nationally representative sample and the ability to explore trends across a range of social, health and demographic factors. Limitations include having only years as time-points, meaning the inability to explore trends using more sophisticated time-series analyses with more time-points. Secondly there were small samples sizes within groups such as ethnicity meaning the inability to explore interaction effects of this variable in detail, and only 3 years data on physical activity, and years when questions on mental health or wellbeing were not asked. Given the wide confidence intervals due to limiting the data to people aged 16 to 24 years, we refrained from interpreting trends in too much detail. Future years’ data will be needed to verify whether trends are increasing or plateauing. Thirdly, correlation does not necessarily mean causation, and further investigation is needed to explore whether common factors are related to widespread decline in drinking among young people. Nevertheless, the use of repeated-cross sectional nationally representative data over the past decade, and the ability to explore drinking patterns and broad trends within sub-groups, has made important contributions to this new area of research.

## Conclusions

Increases in non-drinking were found across sub-groups, including groups less commonly associated with non-drinking. This suggests that this behaviour maybe becoming more acceptable among young people, whereas risky behaviours such as binge drinking may be less normalised; both trends are to be welcomed from a public-health standpoint and should be capitalised on going forward. Smoking and drinking behaviours continue to cluster among young people. Future research should explore attitudes towards drinking and not drinking alcohol among young people.

## Additional file

Additional file 1: Data presented from the Health Survey for England 2005–2015. Supplementary tables include confidence Intervals for **Tables S1-S3** and Multivariable logistic regression on the odds of being a non-drinker versus drinker (**Table S4**) (XLSX 21 kb)

## Abbreviations

BMI: Body Mass Index
GHQ: General Health Questionnaire
HSE: Health Survey for England
IMD: Index of Multiple Deprivation
NS-SEC: National Statistics Socio-Economic Classification
